# Role of Silver Nanoparticle-Doped 2-Aminodiphenylamine Polymeric Material in the Detection of Dopamine (DA) with Uric Acid Interference

**DOI:** 10.3390/ma15041308

**Published:** 2022-02-10

**Authors:** Harjot Kaur, Karamveer Sheoran, Samarjeet Singh Siwal, Reena V. Saini, Adesh Kumar Saini, Walaa F. Alsanie, Vijay Kumar Thakur

**Affiliations:** 1 Department of Chemistry, Maharishi Markandeshwar Engineering College, Maharishi Markandeshwar (Deemed to be University), Mullana, Ambala 133207, India; hk6436929@gmail.com (H.K.); karmsheoran031@gmail.com (K.S.); 2 Department of Biotechnology, Maharishi Markandeshwar Engineering College, Maharishi Markandeshwar (Deemed to be University), Mullana, Ambala 133207, India; reenavohra10@gmail.com (R.V.S.); sainiade@gmail.com (A.K.S.); 3 Department of Clinical Laboratories Sciences, The Faculty of Applied Medical Sciences, Taif University, P.O. Box 11099, Taif 21944, Saudi Arabia; w.alsanie@tu.edu.sa; 4 Biorefining and Advanced Materials Research Center, Scotland’s Rural College (SRUC), Kings Buildings, West Mains Road, Edinburgh EH9 3JG, UK; 5 School of Engineering, University of Petroleum & Energy Studies (UPES), Dehradun 248007, India

**Keywords:** dopamine, silver nanoparticles, cyclic voltammetry, uric acid, electrochemical methods

## Abstract

A viable electrochemical approach for the detection of dopamine (DA) in uric acid (UA) utilizing a silver nanoparticle-doped 2-aminodiphenylamine (AgNPs-2ADPA) electrode was invented. The electrochemical performance of DA showed that the incorporated electrode displayed outstanding electrocatalytic performance to the electrochemical oxidation of DA. In our study, the AgNPs-2ADPA exhibits remarkable catalytic activity, retaining high current value and resilience when employed as a working electrode component for electrocatalytic detection of DA. We have also utilized the bare and polymeric-2ADPA in DA detection for a comparison study. This method offers a facile route with extraordinary sensitivity, selectivity, and strength for the voltammetric detection of DA, even in the presence of UA and ascorbic acid (AA) as interferents, that can be employed for pharmaceutical and biological specimens.

## 1. Introduction

3,4-Dihydroxyphenylethylamine, generally named dopamine (DA), has attracted neuroscientists and researchers since its discovery in the 1950s [1]. Dopamine is a neurotransmitter produced in the central nervous system, that acts by binding to DA receptors. The dopaminergic system plays significant role in various aspects of neuromodulation, like motor control, motivation, reward, cognitive function, maternal and reproductive behaviours. Meanwhile, so many vital functions are reliant on the activation of DA, that it is not shocking that imbalanced DA levels have been implicated in the progression of various neurological disorders, e.g., schizophrenia, Huntington’s disorder, Parkinson’s disorder (the third most widespread one), and even HIV [2,3]. Therefore, the sensitivity and detection of DA in a system are essential for diagnostic purposes [4].

DA is an electroactive and essential neurotransmitter, enabling neuronal transmission in the physiological system as a significant neurochemical [5]. It serves as an extracellular chemical courier in the cardiovascular, renal, hormonal, and primary nervous systems [4]. Various DA analysis strategies, for example, liquid chromatography, spectroscopy, fluorescence and electrochemical characterization, have been employed. However, electrochemical methods are of explicit interest owing to their better sensitivity, short reaction period and cost-effectiveness, and abundance of techniques [6].

Electrochemical sensors have been extensively used to analyze different analytes owing to their lower price [7], high sensitivity, quick response, high selectivity, and easy procedures [8]. Furthermore, techniques using electrochemical sensors doped on non-enzymatic substrates have benefits, for example, high durability, lower price, and good reproducibility than enzymatic analysis approaches. Therefore, to improve the sensitivity of non-enzymatic sensors for DA detection, multiple functionalized electrodes, like graphene (Gr), carbon nanotubes (CNTs), conducting polymers (CPs) materials, and nanostructured metal oxides (MO_x_), offering increased electrocatalytic performance, were used to enhance DA detection [9,10,11].

Significant attention has been focused on silver nanoparticles (AgNPs) as zero-dimensional (0D) nanoparticles because of their premier characteristics: biocompatibility, low toxicity, outstanding electrical and high electrocatalytic performance and a sustainable price [12]. Nevertheless, AgNPs are generally unstable and precipitate in complex fluids due to their strong interatomic energies, resulting in decreased activity and resilience. Thus, 2D materials are appropriate for Ag nuclei development in the absence of aggregation. Lately, various substrates, for example, CNTs, Gr, and even metal sulfides and MOx, have been utilized as platforms for developing Ag NPs. Incorporating AgNPs on organic/inorganic platforms is an adequate procedure to avoid agglomeration that enhances the durability and electrocatalytic performance [13].

In the current study, a silver nanoparticle-doped 2-aminodiphenylamine (AgNPs-2ADPA) electrode was fabricated that exhibited remarkable catalytic activity, retaining high current value and resilience when employed for electrocatalytic detection of DA. The bare and polymeric-2ADPA electrodes were also utilized in DA detection for comparison purposes. This investigation offers a facile route for the voltammetric detection of DA wit extraordinary sensitivity, selectivity, and strength with UA and AA as interferents that can be employed on pharmaceutical and biological specimens.

## 2. Materials and Methods

This report is divided into two parts: characterization of the composite and an assessment of its activity in an electrochemical technique to detect DA. In this study the synthesized materials AgNPs-2ADPA and 2ADPA were applied.

### 2.1. Chemicals and Reagents

All the chemicals and reagents such as silver nitrate (AgNO_3_), methanol (MeOH, ≥99%), sodium borohydride (NaBH_4_, ≥99%), 2-nitrodiphenylamine (C_12_H_10_N_2_O_2_, ≥98%), urea ((NH_2_)_2_CO, ≥99%), ascorbic acid (AA ≥99%), uric acid (UA, ≥99%), ultrapure deionized water (dH_2_O, D.I., 18.25 MΩ), ethanol (C_2_H_5_OH, ≥99%), alumina powder (Al_2_O_3_, ≥99%), ammonium persulfate (APS, ≥99%), sodium dihydrogen phosphate (NaH_2_PO_4_, ≥99%), and dibasic sodium phosphate (Na_2_HPO_4_, ≥99%) were obtained from Merck (St. Louis, MO, USA) and utilized without further purification. A 2.0 M PBS solution (pH = 7.0) was made by blending 62 mL of 2.0 M Na_2_HPO_4_ with 38 mL of 2.0 M NaH_2_PO_4_ solution. Nafion (Nf) perfluorinated resin solution (5 wt.%) was also obtained from Merck.

### 2.2. Electrode Preparation

The electrochemical design utilized a three-probe method containing an anode electrode with 3 mm diameter, a reference (Ag/AgCl), and a counter electrode (CE; platinum wire). The synthesized composites were separately incorporated on the WE (GCE) utilizing a facile approach, and after each individual cycle, the electrode was cleaned and scrubbed. After the washing procedure, the synthesized material was used for the GCE, i.e., incorporating the probe for the subsequent electrochemical analysis. All the electrochemistry-based analyses were done in a cell covered with a lid.

To clean the surface of the WE, different sizes of alumina solutions and ultra-sonication were used for electrochemical rinsing. Then ultimately, to confirm that the GCE exterior was renewed, cathodic rinsing and quick etching through aqua regia were utilized. A flame heated the CE till it was red-hot; after that, it was washed with dH_2_O. Lastly, the RE was permanently kept in a 3M KCl solution when not in use.

The last control was done before the electrode was incorporated and prepared for usage. In this step, the cleaned GCE was placed into a PBS (pH 7.5) solution in an electrochemical system, and a typical CV study was conducted in a voltage range from −0.1 V to + 0.65 V at a sweep rate of 50 mV/s. This electrochemical scan without observing any redox peaks affirmed that the WE was cleaned of all substances and optimized to be employed for the subsequent electrochemical estimation.

### 2.3. Electrochemical Analysis

The electrochemical analyses were conducted in a typical three-electrode system utilizing a Shanghai Chenhua 760 E potentiostat (CHI760E) electrochemical workstation(Shanghai, China). The electrochemical impedance spectroscopy technique (EIS) within the frequency range of 1 MHz to 1 Hz in PBS solution evaluated the synthesized materials’ electrochemical performance. Different methods like cyclic voltammetry (CV); in the potential window from −0.2–0.8 V at a scan rate of 50 mV/s, differential pulse voltammetry (DPV) within a potential window of −0.1 to 0.45 V, and a chronoamperometry (CA) study with an applied voltage of 0.1 V (stable) were conducted to confirm the suitability of the composite for detecting the neuro transmitting analytes and also evaluate its comparative sensitivity and selectivity. Through the different investigation techniques, the bare GCE and polymer-incorporated GCE were utilized as the study’s control elements for the metal, polymeric-doped composites, and guide the identification of the optimized composites.

### 2.4. Material Preparation

Initially, 0.5 M 2-nitrodiphenylamine (2NDPA) used as the original nitro compound for further study was dissolved in MeOH. Subsequently, a small amount of Ag-gC_3_N_4_ composite (5 wt.%) was added to the solution and sonicated for around 20 s to adequately integrate the suspension. NaBH_4_ (0.01 M) was used as a reducing agent for the blend and reacted for around half-hour. Later the composite was removed by percolation, and the monomer unit (2-ADPA) retained. Lastly, the 2ADPA was polymerized through two pathways to design both materials, i.e., (i) polymeric 2ADPA (P[2-ADPA]) and (ii) AgNPs-2ADPA (the AgNP-doped polymerized catalyst). 

The support substance, P[2-ADPA], was produced by polymerizing the monomeric 2-NDPA, using 0.05 M APS as an oxidizing agent. This polymeric material possessed no metallic ingredients in its configuration. Subsequently, AgNPs-2ADPA was prepared through reduction of the 2−NDPA via the slow addition of 0.05 M AgNO_3_ as the oxidizing mediator. The 2NDPA was reduced fully and fashioned an AgNPs-2ADPA polymeric composite.

### 2.5. Material Characterization

The steps of the preparation of the monomeric 2ADPA, P[2ADPA], and AgNPs-2ADPA were observed utilizing the ultraviolet-visible (UV-Vis) spectroscopy (UV 2600; A118757-Shimadzu, Japan) estimation approach. Fourier Transform Infrared (FTIR) spectroscopy (IRSpirit QATR-S; A224057 00293-Shimadzu, Kyoto, Japan) is done to ensure the accuracy of the composite’s formation by relating the FTIR peaks corresponding to the functional groups existing into the produced substance. Transmission electron microscopy (TEM) was performed on the composites to verify their morphology (JEOL, Tokyo, Japan). Ultimately, an EIS investigation was executed to explain the materials’ conductive abilities related to their oxidation potential.

A generalized CV analysis was performed to estimate the operative potential of AgNPs-2ADPA. This method approximated the detection voltage of DA in an electrochemical method with interferents like AA and UA. A further comparison was utilized to decide if an anionic layer will improve the performance of the composite as it could function as an obstacle to delay the scattering of intrusive anions like AA and UA that are understood to have an identical oxidation voltage for DA. Differential pulse voltammetry (DPV) was performed in a cell in which UA was primarily counted, masked by adding growing concentrations of DA. Finally, chronoamperometry (CA) was utilized to observe the effect of continuous addition of DA and interferents to observe the composite’s optimization using an electrochemical approach.

## 3. Results and Discussions

### 3.1. Material Characterization

**Figure 1:**
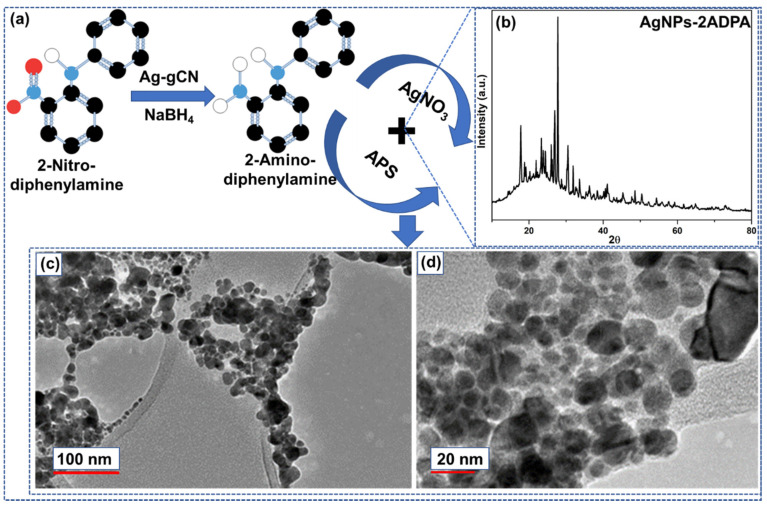
(**a**) Schematic representation of the study to synthesize AgNPs-doped 2ADPA polymeric material. (**b**) XRD pattern of AgNPs-P-2ADPA. TEM pictures of the developed AgNPs-2ADPA composite i.e., (**c**) (100 nm) and (**d**) (20 nm).

Figure 1a shows an overview of the synthesis process of the proposed material, i.e., AgNPs-doped-2ADPA. The nanoparticles prepared through this approach were examined utilizing powder XRD to verify the silver particles’ structural details. Figure 1b shows the XRD pattern of AgNPs-poly-2ADPA. In this case the peaks that occurred were weaker compared to silver. It can be due to the bioorganic combinations appearing on the exterior of the AgNPs [14]. For the comparison, the XRD spectra for P-2ADPA, gC_3_N_4_ and Ag-gC_3_N_4_ have been provided in Figures S1–S3 in the Supplementary Information, respectively. The TEM images (Figure 1c,d) indicated the interior structure of the AgNPs-2ADPA composite. The black dots in the picture are the AgNPs doped into the polymeric sheet. High magnifications showed the elevated-density spread of the shady spots and demonstrated that the Ag metallic atoms were within the nanoscale region.

**Figure 2:**
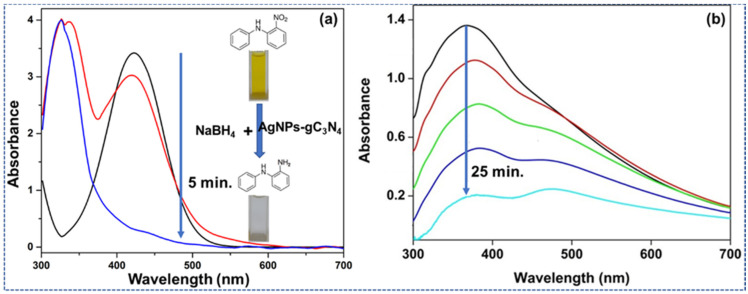
(**a**) UV-Vis spectrum of the appearance of monomeric 2ADPA from 2NDPA. (**b**) UV-Vis spectrum of the polymerization procedure of the 2ADPA into AgNPs-2ADPA-doped polymer afterward introducing the AgNO_3_.

Figure 2a shows the UV-Vis spectrum of the generation of monomeric 2ADPA through 2NDPA. This implicated the removal of 2NDPA, including NaBH_4_ (the source origin of H-), with an AgNPs-gC_3_N_4_ composite. A wide-ranging peak around 435 nm is quenched as the monomer is formed. The decrease of the 2NDPA curve is due to the incremental removal of the nitro groups and confirms the appearance of the 2ADPA monomeric unit. After adding AgNO_3_ into 2ADPA, a quick polymerization procedure was started (Figure 2b). The AgNO_3_ enhanced the removal rate of monomer owing to the appearance of AgNPs, which catalyzed the ET-PT mechanism. Here, we demonstrate that two separate oxidation and reduction responses emerge within the same reaction vessel. The oxidation of 2ADPA (polymerization) directs towards the reduction of AgNO_3_, an instance of a sequential electron−proton- transfer (EPT) mechanism. Additionally, the focus is provided on the reduction method catalyzed by AgNPs. The polymerization was rapid while the AgNO_3_ oxidizing mediator was added and attained a polymerization peak after 25 min. A small peak is noticed at 315–320 nm owing to the π-π* transition occurring during the partial oxidation [15,16]. For comparison, the UV-Vis spectrum of Ag-gC_3_N_4_ is supplied in the Supplementary Information (Figure S4) [17]. After incorporating silver nanoparticles (Ag-gC_3_N_4_), the substance showed lower absorbance within the visible region. The nickel NPs have less absorbance in the visible area, reducing the absorption shape under the visible part.

At 360–380 nm, the central peak was reduced with time and moved towards a higher wavelength. This area distinguishes the polaron-bipolaron evolution and states owing to the appearance of polymer. The broadening of the peak towards the right side demonstrated the formation and development of the NPs in the configuration [18]. The wide peak around 470–475 nm slowly rises due to benzenoid to quinoid excitonic growth. This transition affected a high energy band by partially containing oxidation highest occupied molecular orbital towards complete oxidation lowest unoccupied molecular orbital (HOMO to LUMO). It appeared owing to the quinoid ring formation of the composite. If the composite has quinoid rings, the electrons (e^−^) and protons (H^+^) will not be as sensitive if they remain in a stable condition. It would result in a lower effective recognition of DA.

**Figure 3:**
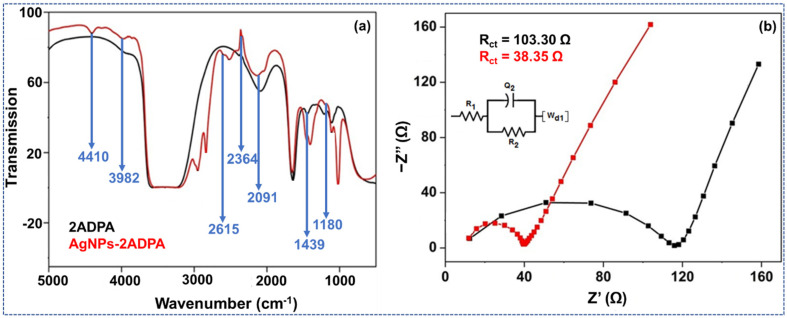
(**a**) FTIR spectra to investigate the functional groups and distinctive vibrations in both composites, i.e., P-2ADPA (black curve) and AgNPs-2ADPA (red curve). (**b**) An EIS analysis of P-2ADPA (black curve) and AgNPs-2ADPA (red curve) in PBS medium.

A Fourier transform infrared (FTIR) spectroscopic approach was used to study the optical nature of the materials produced: 2ADPA and AgNPs-2ADPA. The diagrams were superimposed as a standard for comparing the pristine and polymer-doped products. Figure 3a shows the typical peaks within the composite. The broad peak around 3400 cm^−1^ conforms to the expected stretching vibrations of NH or NH assemblies that are amine clusters. The peak around 2991 cm^−1^ commonly develops in this area for single bonded amines, including N-H bonds. This peak became visible within the doped metal composite. The AgNPs deepen the infrared plasmon excitations and enhance the spectral sensitivity, perhaps due to the exterior-doped IR absorption [19]. A typical stretching vibration of the quinoid configuration is exhibited around 1645 cm^−1^. A CN stretching mode is exhibited around 1439 cm^−1^; this seems low when the nitro cluster is conjugated through a benzene ring. 

Furthermore, the N_2_-comprising clusters are determined through formation of two influential bands around 1645 and 1439 cm^−1^. An aromatic C-H in-plane bending vibration appears around 1109 and 1040 cm^−1^. Finally, the metallic particles induced a redshift due to the decreased bond order due to the appearance of metal-nitrogen bonds [20]. The complex spectrum showed the appearance of pristine and or strengthened peaks at 1439, 2836, 2958, 3982, and 4410 cm^−1^ owing to the formation of M-N interactions. It indicates P-2ADPA formation as the FTIR spectra possess the peaks related to the network and that the AgNPs existed in a chain form that particularly intensified the spectra. The subsequent TEM study examined the internal morphology to ensure the formation of AgNPs that were incorporated upon the polymeric chain, and were in the nano-scale range.

The oxidation voltages of both composites was estimated utilizing EIS examination under ambient conditions (Figure 3b). The AgNPs-2ADPA design can be assumed to be a single parallel *Q*_2_ and *R*_2_ matching circuit connected with the Warburg diffusion coefficient term (*W_d1_*). The more elevated conductivity of the AgNPs-2ADPA substitutes for the electronic assistance of polymer and delivers further impact to the *W_d1_* in the substance. The electron transfer resistances of P-2ADPA and AgNPs-2ADPA were 103.30 Ω and 38.35 Ω, respectively, associated with the electron transfer resistance of the redox electrode liquefied with the electrolyte media. The augmented metallic composite had a much lower resistance than the P-2ADPA due to the acceleration of the redox reaction. This revealed that AgNPs-2ADPA was more catalytically responsive than the P-2ADPA [21]. The boost in electric performance of AgNPs-2ADPA was enhanced by the metal NPs incorporated all over the composite. These metal NPs enhanced the surface area where the redox reaction would occur [22]. The description analysis approaches demonstrated that P-2ADPA and AgNPs-2ADPA composites were formed and their electrochemical performance for DA could be examined. The results reveal a semicircle in the upper-frequency area and an unbent part within the lower frequency area. The semicircle portion within the Nyquist plot illustrates the Faradaic charge transfer operation. The interfacial charge transfer resistance was instantly estimated from the diameter of the semicircle.

### 3.2. Electrochemical Studies

Electrochemical techniques are valuable due to their unsophistication, economy, speed, better sensitivity and easy optimization. DA oxidation occurs upon the WEs’ surface. This is supposed to accumulate on the WE’s surface’s multilayer and afterwards yields an e^−^ towards the probe exterior as it is oxidized [23]. When target biomolecules are examined via a sensing substance deposited upon the anode electrode of an electrochemical biosensor, in the matter of DA, multiple electrochemical techniques (such as CA, CV, DPV) have been created since DA may be oxidized readily, leading to the appearance of dopamine-*o*-quinone via a two-electron method [24]. The electrons liberated via DA during its oxidation induce currents, which can be linear based upon the absorption of the electroactive DA biomolecules, allowing quantifying these mixtures. Electrochemical approaches have numerous advantages for DA detection: the low cost of electrochemical instrumentation, the size of the electrodes, which may be conveniently embedded within living cells, the short response period, and the capability to observe DA in real-time.

#### 3.2.1. Cyclic Voltammetry (CV)

CV is a compelling technique utilized for redox-active substrates. This is the preliminary stage to select the possible recognition of analytes through diverse incorporated forms of the WE. The incorporations possess easy P-doped electrodes and layers showing ionic wave discrimination. As a ionic substance, DA has been sensed utilizing films showing cationic permselectivity or just P-doped probes [25].

Superficial P-doped probes have latent ability for determining analytes by not using permittivity. They can detect DA and anionic interferents like AA and UA concurrently if it is a better separation of their corresponding redox voltages. An anionic layer like Nafion (Nf) would hinder the dispersal of inhibiting anionic ions, for example, AA, UA, and therefore selectively detect DA as a cationic species at a suitable pH [26]. The following diagrams studied the composites, and their voltage oxidative responses for all the analytes.

The oxidation of DA occurs on the exterior of the WE. The surface of the WE was modified with both composites to enhance the NT oxidation. The second analysis covered these incorporated electrode shells with an anionic material—Nf. This layer is explicitly concentrated upon the ionic essence of DA and its accompanying conductive species, AA and UA. DA, AA, and UA possess identical oxidation voltages and interfere with practical DA detection. The ionic behaviours of these analytes offer a dual-class of change to confirm that effective discriminative detection of DA takes place in the presence of AA and UA; where Nf will work as a discriminating factor [27]. The recognition can either take place with AgNPs-2ADPA or Nf-modified-AgNPs-2ADPA. If the first composite cannot successfully differentiate among DA and the interferents, the second composite could produce an ionic hindrance for AA and UA to effectively distinguish between DA and the interferents.

The supporting electrolyte is vital to the electro-organic response. The following facts are essential when choosing a supporting electrolyte: (i) solubility in the solvent typically utilized for the electrolysis; (ii) electrochemical steadiness; (iii) interchange through the reaction medium; and (iv) comparative complexity of the procedure.

Solvents like water, methanol and dimethylformamide dissolve various inorganic supporting electrolytes, where only organic supporting electrolytes are employed in organic solvents. The kinetic study demonstrates that the essence of the electrolyte has a practical impact on the OER/EOP operations, particularly within a high overpotential environment. Tafel coefficients are conditional at the essence of the electrolyte, heat, and overpotential, and the values support preliminary water release as rds. The unnatural effect of the heat upon the Tafel coefficient and transfer coefficient is attributed to the consequence of anion adsorption and driblet adherence at the electrode/electrolyte interface [28]. The objective of the supporting electrolyte is to improve the ion exchangeability of the solution and eradicate the electric domain from the electrolyte.

**Figure 4:**
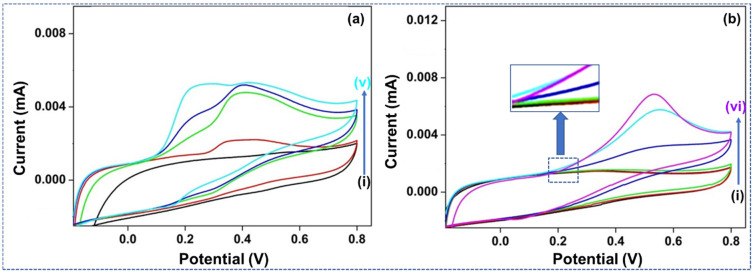
(**a**) CV study of GCE into PBS. (i) Without any analyte while (ii)–(iv) are the separate analytes added to the electrolyte. (**b**) CV study of GCE, doped with Nf, within PBS. (i) Without analyte, whereas (ii)–(vi) are the separate analytes added to the electrolyte.

Figure 4a,b shows the CV study of bare and the Nf-doped bare GCE, in (i) without and (ii)–(v) with the individual analytes. Their reactions were analyzed as undoped and doped forms, including Nf to DA, AA, and UA. The undoped WE (Figure 4a) reacted to the increase of AA and UA (100 and 60 µM) by forming oxidation signals around 0.35 and 0.41 V. The broad, irreversible rises represent the sluggish e^−^ transmission mechanisms of the DA utilizing the GCE. For example, when 4 and 20 µM DA amounts were added, another peak evolved around 0.2 V in the vessel. This reaction indicated that the undoped GCE could respond to the DA and interferents—AA and UA—concurrently; meanwhile, a better peak split was created between their redox voltages. Anodic peak overlapping occurs the potential capacity region from (−0.2–0.6 V). Ideally, three well-observed CV crests would have formed. However, the study created different oxidation peaks that permitted us to determine the oxidation voltage connected to each individual analyte.

Figure 4b represents the reaction of a GCE doped by Nf within a PBS electrolytic media, where (i) is without and (ii)-(vi) are with the individual analytes—expanding (ii) UA and (iii) AA (both 100 µM) added without an oxidative reaction. It demonstrates the impact of the anionic Nf as a layer upon the GCE for anionic analytes like AA and UA. These analytes were repulsed by the charge formed at the exterior of the GCE and would their oxidative responses would not endure to deliver a potential reaction. The DA-based analytes were continuously oxidized at about 0.5 V. Their raised concentrations (4, 12, and 20 µM DA) in the solution generated a high reaction current.

#### 3.2.2. CV Study Employing the P-ADPA-Doped Electrode

**Figure 5:**
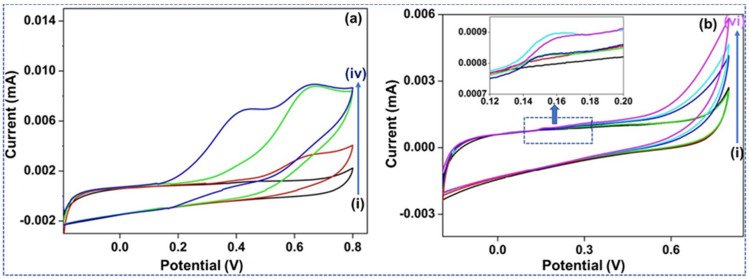
(**a**) CV study of 2ADPA in PBS while (i) is without any analyte where (ii)–(iv) are with analyte mixed into the electrolyte. (**b**) CV study of 2ADPA, Nf-doped into PBS while (i) is without any analyte where (ii)–(vi) are with corresponding analytes mixed into the electrolyte.

The CV study of the 2ADPA within PBS at (i) without the separate analytes, and (ii)-(iv) with analyte are illustrated in Figure 5a. Primarily, as seen in (ii) 160 µM UA was mixed into the electrolyte, afterward (iii) 100 µM AA was added into the electrolyte, and then finally (iv) 32 µM DA was mixed into the electrolyte. A substantial peak was formed at 0.65 and 0.6 V in response to both intrusive agents: AA and UA. When DA was mixed into the cell, another oxidation peak formed around 0.4 V. This confirms that the straightforward P-2ADPA may sense DA, AA, and UA simultaneously. While considering the formation of the peaks, it was apparent that the e^−^ transmission mechanisms were lagging owing to the vast peak size. The peaks shift towards the higher potential for all analytes revealing that comparing a polymeric chain to the bare GCE, the former reduces the catalytic performance of this procedure.

Figure 5b illustrates the CV study of the 2ADPA material, Nf-doped, in PBS where (i) is with and (ii)–(vi) are without the separate analytes. Primarily, (ii) UA and (iii) AA (both 100 µM) were mixed with the electrolyte without any oxidation signal. With increasing concentrations of DA, i.e., 4, 20, and 32 µM DA, an oxidative response was created and a peak started to appear around 0.6 V. The Nf-doped P-2ADPA GCE incorporation formed a design capable of discriminating for DA recognition and stopped the anionic interferences.

The CV cycles of all the strategies stated previously (GCE and P-2ADPA, non-doped and doped) delivered definitive oxidation signals but no equivalent well-defined reduction signals. This indicates that they are inadequate procedures in respect to the electrochemical performance. The following paragraph explains how the metal incorporated polymer has the potential to provide a large surface area, including many active sites to enhance the e^−^ transmission between the probe and the analytes, focused explicitly on the DA [21].

#### 3.2.3. CV Study Utilizing the AgNPs-2ADPA Doped Electrode

**Figure 6:**
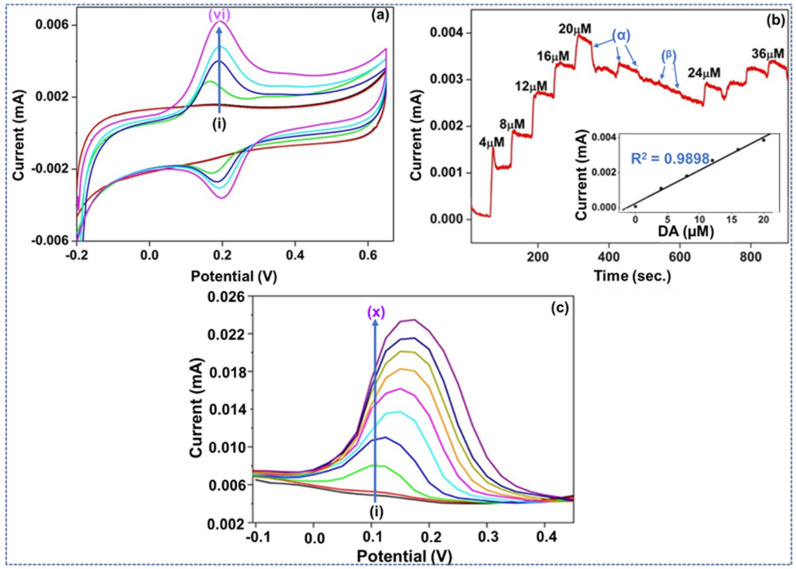
(**a**) CV study of Nf-doped AgNPs-2ADPA, in PBS where (i) is without any analyte and with (ii) 140 μM UA and (iii)–(vi) the consecutive additions of DA. (**b**) CA effects of the AgNPs-2ADPA device after consecutive additions of DA (4–20 µM DA) into PBS. (α) and (β) show the steps at which exact interferents were added to the cell, including UA and AA. (**c**) DPV analysis of Nf-doped AgNPs-2ADPA, in the (i) without and with (ii) 140 μM UA interference and (iii)–(x) 4–32 μM DA.

The CV study of AgNPs-2ADPA, Nf-doped into PBS is illustrated in Figure 6a, where (i) is without and (ii)–(vi) with different analytes. Throughout the addition of 140 μM UA, no signal indicated an oxidation response. However, when subsequently 4, 12, 20, and 32 μM DA was added completed. The design produced an ideal redox response curve with a potential discrepancy around 0.2 V. The current persisted as the DA addition increased, indicating a linear effect of the addition of DA. Besides, the Nf layer prompted a flourishing discriminative effect among the interferents and DA. The Nf-doped probe surface acted as a binder and a discriminatory ionic component that formed a repelling anionic state, blocking analytes like UA and AA from reaching the electrode exterior.

With and without an Nf coating, all three composite additions sensed DA in lower absorptions (4–32 μM) while its evaluated activity-dependent concentration is around 1.6 mM. Regarding the most important factors of a CV study, the AgNPs-2ADPA surpassed the other composites. Nf-doped AgNPs-2ADPA comprised two oxidation and reduction pairs showing better electrochemical performance. At the same time, GCE and P-2ADPA (without and with Nf-doping) possessed no peak resembling a well-defined reduction signal. Nf-doped AgNPs-2ADPA also showed an enhanced electrocatalytic performance, as a more increased peak current was formed during the addition of 4 µM DA (compared to the Nf-covered techniques). The more elevated oxidation peak current is strictly associated with an improved oxidation rate owing to the enriched nature of the composite. This is due to the metal NPs, which grow the surface area accessible to the oxidation of DA. Finally, the peak split declined as the material activity improved. Overall, improved catalytic performance for DA is contemplated with a lower oxidation voltage and a higher current compared to the Nf-based AgNPs-2ADPA composite [29]. The cathodic presence represents the reduction of the substrate present on the electrode surface, so it further confirms the formation of silver nanoparticles within the system responsible for enhancing the catalytic performance of the proposed material.

The amperometry approach (differential pulsed) constantly reacted, with a steady applied voltage, rising into the current with subsequent of 4 μM DA in the PBS electrolytic media, as illustrated in Figure 6b. With the addition of AA and UA under the optimized testing conditions as potentially interfering components, the AgNPs-2ADPA incorporated electrode showed good non-participation of AA and UA. This is essential to indicate that the selectivity for DA occurred without the Nf layer.

The insert figure illustrates a standardization curve where the successive currents reacted to sequential additions of DA. This calibration curve was shown to display a correlation coefficient of R^2^ = 0.9898. The linear response indicated the composite’s resilience for the growing DA addition. The linear relationship correlation coefficient (R^2^) of more than 0.98, indicates a Faradaic processes [30].

After adding the interferents, DA was furthermore added in the procedure. The composite continued to respond to the addition of DA and thus its catalytic performance did not deteriorate, affirming the selective nature to detect DA with AA and UA present [31].

Using the DPV detection technique DA was studied by observing the current value of DA with UA as a named biological interferent. The current values were parts of the potential used for the electrode and constant scan rate. UA produce a minimal response, and its outcome was insignificant. Patella et al. [32] also reported a minor reaction for UA and AA in the DPV analyses since they produced around 12- and 6% responses. AgNPs-2ADPA was capable of enhancing the catalytic performance of DA. DA was sensed at low absorptions beginning from 4 µM in the cell. Figure 6c illustrates the continual growth in current with increased DA concentration. AgNPs-2ADPA produces an ideal differentiation of a catalytic composite by growing with its current oxidation performance. DA generally has an oxidation voltage range from 0.3–0.4 V upon bare and incorporated GCE. In this example, the peak voltage of DA is nearly 0.2 V. The electrochemical performance discrepancy is due to the presence of metal NPs (MNPs) that improve the removal of the electrons of DA, which improves the oxidation current and causes the oxidation voltage value to decline [33]. The polymer-doped AgNPs-2ADPA also directly impacts the catalytic performance of the synthesized composite by decreasing the reaction overpotential of the P-2ADPA owing to its electronic response. DPV showed that DA could be continually sensed at a low concentration and produce a proportional current value. Nf-doped AgNPs-2ADPA is an advantageous medium to sense DA at low concentrations in a potential range of −0.1 to 0.4 V. CA was used finally to examine the selectivity of AgNPs-2ADPA in particular.

## 4. Conclusions

In this report we have successfully synthesized a polymeric AgNPs-2ADPA composite that can act as a neurotransmitter. This polymeric nanomaterial offers a practical, selective, and discreet sensing procedure that can successfully detect DA at low concentrations in a PBS dependent system. The metal NP offered significant conductive possibilities to the polymeric material when referring to the EIS spectra (i.e., the *R_ct_* values for P-2ADPA and AgNPs-2ADPA are 103.30 Ω and 38.35 Ω, respectively). The MNPs have a promising effect upon DA recognition owing to their enabling charge transport via the P-2ADPA. The incorporation of AgNPs in the polymeric sheet generated an adequate charge bouncing result, which is further confirmed by the CV analysis when we have consecutively added DA (4, 12, 20, and 32 μM). The electronic transfer between the redox pair and the MNP encouraged a drop of the oxidation peak of the DA, noticed during the electrochemical study of the synthesized composites with DA.

## Data Availability

Not applicable on our study.

## Supplementary Materials

The following supporting information can be downloaded at: https://www.mdpi.com/article/10.3390/ma15041308/s1, Figure S1: XRD pattern of P-2ADPA; Figure S2: XRD pattern of gC_3_N_4_; Figure S3: XRD pattern of Ag-gC_3_N_4_; Figure S4: UV−vis spectrum of Ag-gC_3_N_4_.
