# Supplementary material for: Role of Silver Nanoparticle-Doped 2-Aminodiphenylamine Polymeric Material in the Detection of Dopamine (DA) with Uric Acid Interference
Source: Materials (Basel). 2022 Feb 10;15(4):1308. doi: 10.3390/ma15041308 (PMC8875575; doi:10.3390/ma15041308)
Supplement: Supplementary file 1 [file materials-15-01308-s001.zip › materials-1557363-supplementary.pdf]

*Article*

# Role of Silver Nanoparticle-Doped 2-Aminodiphenylamine Polymeric Material in the Detection of Dopamine (DA) with Uric Acid Interference

**Harjot Kaur [1], Karamveer Sheoran [1], Samarjeet Singh Siwal [1,*], Reena V. Saini [2], Adesh Kumar Saini [2], Walaa F. Alsanie [3] and Vijay Kumar Thakur [4,5,*]**

1 Department of Chemistry, Maharishi Markandeshwar Engineering College, Maharishi Markandeshwar (Deemed to be University), Mullana, Ambala, 133207, India; hk6436929@gmail.com (H.K.); karmsheoran031@gmail.com (K.S.)
2 Department of Biotechnology, Maharishi Markandeshwar (Deemed to be University), Mullana, Ambala, 133207, India; reenavohra10@gmail.com (R.V.S.); sainiade@gmail.com (A.K.S.)
3 Department of Clinical Laboratories Sciences, The Faculty of Applied Medical Sciences, Taif University, P.O. Box 11099, Taif 21944, Saudi Arabia; w.alsanie@tu.edu.sa
4 Biorefining and Advanced Materials Research Center, Scotland's Rural College (SRUC), Kings Buildings, West Mains Road, Edinburgh EH9 3JG, UK;
5 School of Engineering, University of Petroleum & Energy Studies (UPES), Dehradun 248007, India
* Correspondence: samarjeet6j1@gmail.com (S.S.S.); Vijay.Thakur@sruc.ac.uk(V.K.T.)

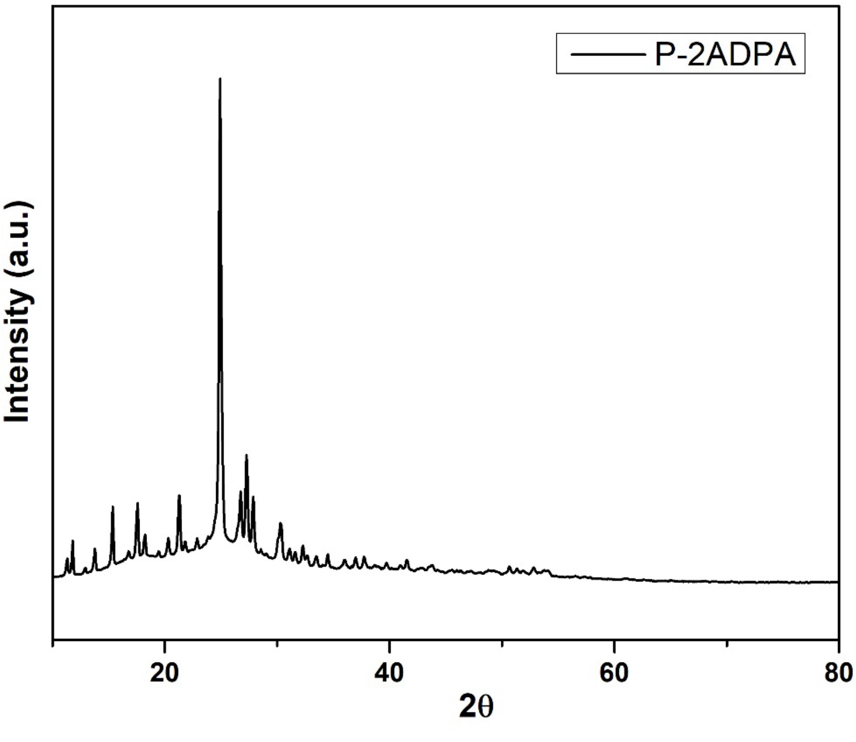


**Figure S1.** XRD pattern of P-2ADPA.

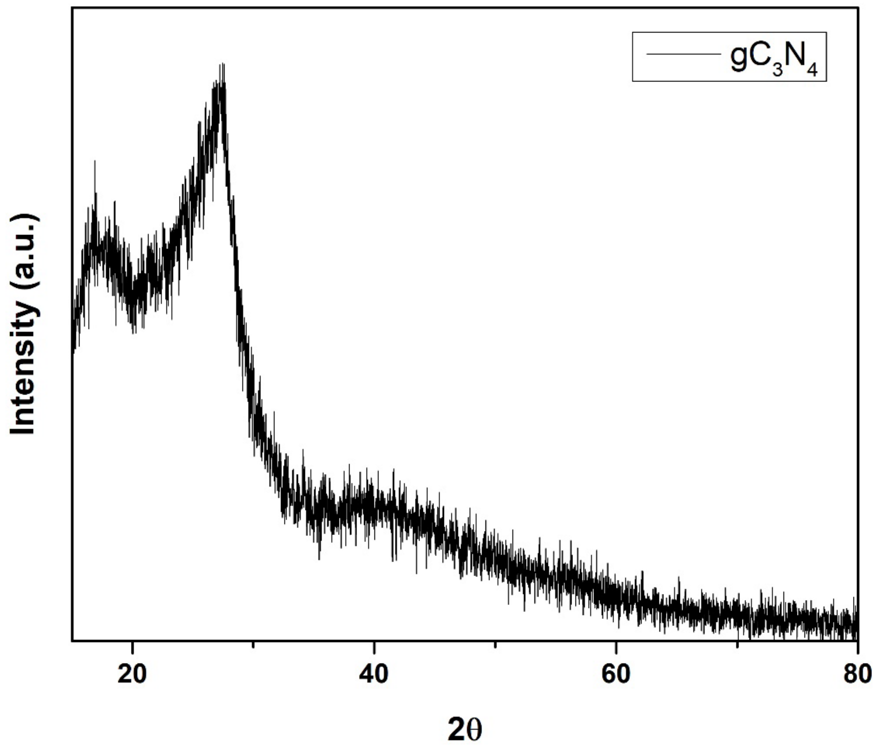


**Figure S2.** XRD pattern of $gC_3N_4$.

**Figure S2** indicates the XRD pattern of the $gC_3N_4$, besides the carbon nitride peak at 27.33°.

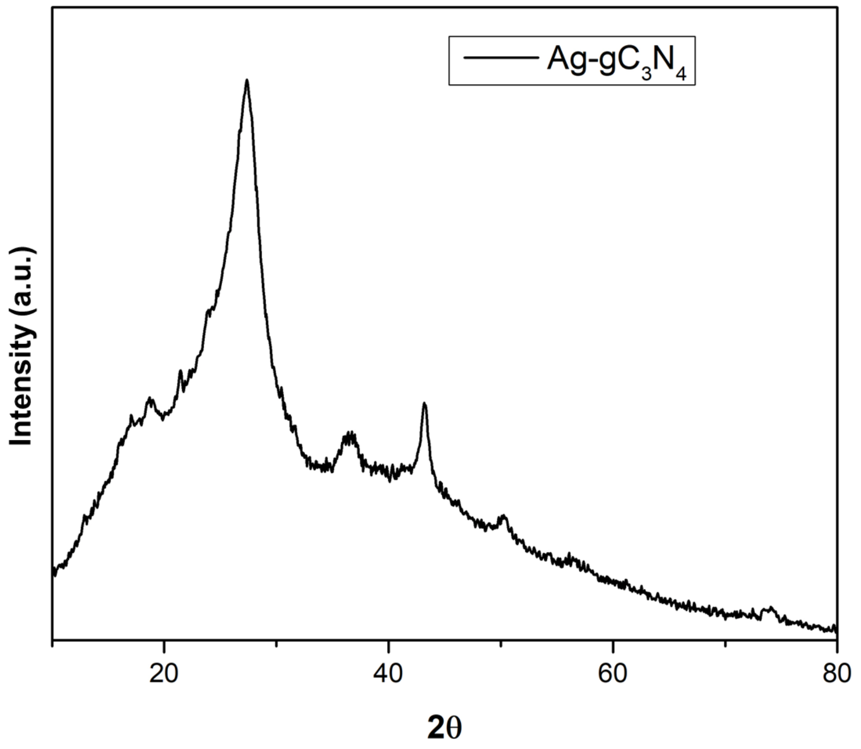


**Figure S3.** XRD pattern of Ag-g$C_3N_4$.

**Figure S3** indicates the XRD pattern of the Ag-g$C_3N_4$; besides the carbon nitride peak at 27.33°, the other four peaks at 40.10°, 46.0°, 68.40, and 82.16° can be assigned to the metallic silver.

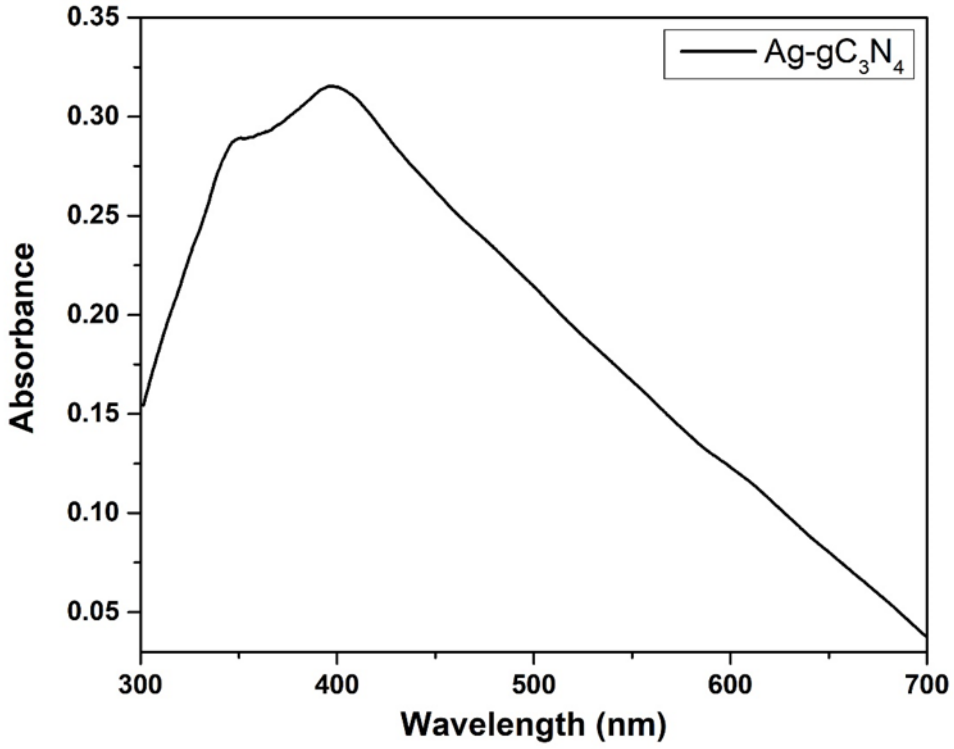


**Figure S4.** UV-vis spectrum of Ag-g$C_3N_4$.
